# Supplementary material for: Systemic Treatment Selection for Patients with Advanced Pancreatic Neuroendocrine Tumours (PanNETs)
Source: Cancers (Basel). 2020 Jul 21;12(7):1988. doi: 10.3390/cancers12071988 (PMC7409353; doi:10.3390/cancers12071988)
Supplement: Supplementary file 1 [file cancers-12-01988-s001.pdf]

Supplementary Materials: Systemic treatment selection for patients with advanced pancreatic neuroendocrine tumours (PanNETs)

Vera G. Megdanova-Chipeva, Angela Lamarca , Alison Backen, Mairéad G. McNamara, Jorge Barriuso, Sonia Sergieva, Lilia Gocheva, Was Mansoor, Prakash Manoharan and Juan W Valle

Table S1. Summary of studies exploring the role of SSAs.

Study	Design	Treatment	Patient selection (Grade, Prior treatment, ECOG PS)	N	Primary End Point	median PFS (months)	median OS (months)	DoR (months)	ORR (%)	DCR (CR+PR+SD) (%/n)	Grade3–4 toxicity (%)
PRO MID [21,165]	Phase IIIb Placebo-controlled, double-blind, prospective, randomised study<br><br>* Pts on placebo crossover after progression on active arm;	Octreotide LAR 30 mg vs Placebo	G1<br>Locally advanced or metastatic Midgut NET or with unknown origin, treatment naïve;<br><br>Karnofsky >/=80%<br>(86% of study population)	85 pts<br><br>Octreotide 30 mg = 42 pts<br>Placebo = 43 pts	TTP	Time to tumour progression:<br><br>Oct = 14.3 m<br>(95% CI, 11.0 to 28.8 m)<br>Pl=6 m,<br>(95% CI, 3.7 to 9.4 m)<br>([HR] = 0.34; 95% CI, 0.20 to 0.59; *p* = .000072)	Oct 84.7 m<br>Placebo 83.7 m<br>[HR = 0.83 (95% CI: 0.47–1.46); *p* = 0.51]<br>*median OS from diagnosis – 106 m<br><br>5 y survival 67%<br>10-y survival 46%	NA	1% vs 1%	Oct 30 mg<br>CR = O<br>PR = 1<br>SD = 28<br><br>Placebo<br>CR = 0<br>PR = 1<br>SD = 16	GI tract 6 vs 8<br>Haematological 5 vs 1<br>Fatigue/Fever 8 vs 2
Jann, 2013 [59]	Retrospective	Octreotide LAR	G1-2, unknown<br><br>Advanced F or NF PanNET<br><br>ECOG PS- NS	43 pts	ORR, DCR, ORR and DCR at 12 months, TTP	TTP – 13 m<br>*median TTP for Ki67 >10% compared with Ki67 <5% (*p* = 0.009) and Ki67 5-10% (*p* = 0.036)	98 m	NA	7%<br><br>ORR at 12 m<br>5%	65%<br>PR 7% (*n* = 3)<br>SD 58% (*n* = 25)<br><br>DCR at 12 m<br>PR 5% (*n* = 2)<br>SD 37% (*n* = 16)	NS

Table S1. *Cont.*

CLARINET [16,17,60,61]	Phase III randomised, double-blind, placebo-controlled, 96-week study followed by an open label extension (OLE) part<br><br>*Crossover of pts on placebo after progression on active arm;	Lanreotide autogel 120 mg vs Placebo	G1/2<br>(Ki-67 </= 10%)<br><br>Locally advanced or metastatic, non-functioning (except grastrinomas) GEP-NETs, received or not prior Tx, SSR +<br>*Basline stable disease<br>96% in Lan vs 95% in Pl-arm<br>** Tx-naïve pts – 84 % in both arms<br>ECOG PS NS	204 pts<br><br>PanNET = 91<br>Lan vs Pl<br>42 vs 49 pts<br><br>OLE = 88 pts<br>PanNET = 42	PFS<br><br>OLE<br>+ Safety	Lan vs PL<br>Not reached vs 18.0 m<br>(95% CI 12.2–24.0).<br>HR 0.47 (95% CI 0.30–0.73);<br>*p* < 0.001<br>PanNET<br>HR 0.58<br>(0.32–1.04)<br>Core study<br>30.8 m (95% CI: 30.0; 31.3).<br>Core/OLE study<br>38.5 m (95% CI: 30.9; 59.4]<br>PanNETs<br>(Core/OLE)<br>29.7 m [95% CI:12.0; 38.5]	Not reported	NA	2% vs 0%	Core study[60]<br>Lan 66%<br>CR 0% (*n* = 0)<br>PR 2% (*n* = 2)<br>SD 64% (*n* = 65)<br><br>Placebo 43%<br>CR 0% (*n* = 0)<br>PR 0% (*n* = 0)<br>SD 43% (*n* = 44)	Lan vs Pl<br>25 vs 31%<br><br>Diarrhoea 26 vs 9%<br>Abdominal pain 14 vs 2%<br>Cholelithiasis 10 vs 3%<br>Flatulence 8 vs 5%<br>Injection-site pain 7 vs 3%<br>Nausea 7 vs 2%<br>Vomiting 7 vs 0%
Cives, 2015[62]	Phase II open-label study	Parsireotide LAR	G1-2<br><br>Treatment-naive patients with metastatic NET<br>ECOG 0-1	29 pts<br><br>PanNET = 6 pts	PFS<br><br>PanNET - NS	11 m<br><br>PanNET - NS	NR<br>30-month - 70%<br>PanNET-NS.	NS	4%<br><br>Pan NETs-NS	64%<br>PR 4% (*n* = 1)<br>SD 17% (*n* = 60)<br>PanNETs - NS	All<br>Hyperglycaemia<br>Cholecystitis

N—number median PFS—median progression free survival median OS—overall survival DoR—duration of response ORR—objective response rate DCR—disease control rate G—grade pts—patients TTP—time to tumour progression NET—neuroendocrine tumours Oct—octreotide LAR Pl—placebo HR—hazard ratio CI—confidence interval CR—complete response PR—partial response SD—stable disease NA—not applicable GI—gastrointestinal NS—not stated Lan—Lanreotide autogel # Investigator assessment *BICR (blinded independent central review) assessment / PanNET —pancreatic neuroendocrine tumours F-PanNET—functioning pancreatic neuroendocrine tumours NF-PanNET—non-functioning pancreatic neuroendocrine tumours OLE—open label extension GEP-NET—gastro-enteropancreatic neuroendocrine tumours.

Table S2. Summary of studies exploring the role of targeted therapies.

Study	Design	Treatment	Pts Selection (Grade, Prior Tx, ECOG PS)	N	Primary End Point	Median PFS (Months)	Median OS (Months)	DoR (Months)	ORR (%)	DCR (CR+PR+SD) (%/n)	G3-4 Toxicity (%)
**RADIANT-1 [22]**	Phase II<br>open-label, nonrandomised study, stratified by<br>ongoing SSA at study entry	Everolimus	Well/Moderately differentiated<br><br>Metastatic PanNETs progressed on or after CHT	160 pts<br><br>-SSA<br>N = 115<br>+SSA	ORR in the group without SSA	-SSA<br>& 9.7 m<br>(95% CI,<br>8.3 to 13.3 m)<br># 8.5 m	- SSA<br>& 24.9 m<br>(95% CI,<br>20.2 to 27.1 m)	-SSA<br>& 10.6 m<br>(95% CI, 9.8 m-NA)	-SSA<br>& 9.6%<br>(95% CI<br>4.9-16.5%)	-SSA<br>& 77.4% /*n* = 89/<br>PR 9.6% /*n* = 11/	- vs +SSA<br>Asthenia 5.2vs2.2%<br>>Glucose 4.3vs4.4%<br><Thrombocytes 2.6vs8.9%

			ECOG PS 0-2	N = 45		(95% CI,<br>7.8 to 11.8 m)<br>+SSAs<br>& 16,7 m<br>(95% CI,<br>11.1 to NA)	# NS<br><br>+SSA<br># NR (95% CI,<br>23.0 to NA)	# 19.2 m<br>(95% CI 5.3 to NA)	#10.4% (95% CI, 5.5- 17.5%)	SD 67.8% /*n* = 78)<br><br># 72.2% /*n* = 83/<br>PR 10.4% /*n* = 12/<br>SD 61,7% /*n* = 71/<br><br>+SSA<br>& 84.4% /*n* = 38/<br>PR 4.4% /*n* = 2/<br>SD 80% /*n* = 36)	Stomatitis<br>Diarrhoea<br>Fatigue<br>Anaemia	4.3vs2.2%<br>3.5 vs 0%<br>4.5 vs 0%<br>4.3 vs 4.4%
RADIANT-3 [22,24,25,124]	Ph III<br>prospective, randomised, placebo-controlled, double blinded study;<br><br>Cross-over at progression to open-label Eve	Everolimus vs Placebo	G1/2<br><br>Metastatic PanNETs progressed on prior Tx<br><br>ECOG PS 0–2	410<br><br>Eve<br>N = 207<br>Placebo<br>N = 203	PFS	EVE vs PL<br><br>#11 vs 4.6m<br>HR 0.35;<br>95% [CI], 0.27 to 0.45; *p* < 0.001<br><br>&11.4 vs 5.4 m HR 0.34; 95% CI, 0.26 to 0.44; *p* < 0.001	Eve vs PL<br><br>44.0 vs 37.7 m<br><br>HR 0.94;<br>95% CI, 0.7 to 1.2), *p* =0.30	NA	No Prior CHT:<br>Eve 4,.%<br>(*n* = 5.95% CI 1.6–10.9)<br>Placebo<br>2,0%<br>(*n* = 2.95% CI 0.2–6.9) #<br><br>Prior CHT:<br>Eve 4.9%<br>(*n* = 5.95% CI 1.6–11.0)<br>Placebo<br>2.0%<br>(*n* = 2 .95% CI 0.2–7.0) #	No Prior CHT:<br>Eve 77.9% /*n* = 81/<br>PR 4.8% /*n* = 5/<br>SD 73.1% /*n* = 76)<br><br>Pl 47.1% /*n* = 48/<br>PR 2% /*n* = 2/<br>SD 45.1% /*n* = 46/<br><br>Prior CHT:<br>Eve: 77.7% /*n* =80/<br>PR 4.9% /*n* =5/<br>SD 72.8 /*n* = 75)<br><br>Pl 58,4% /*n* = 59/<br>PR 2% /*n* =2/<br>SD 56.4% /*n* = 57/	Infections<br>Pneumonitis<br>Hyperglycaemia<br>Stomatitis<br>Anaemia	Eve vs Pl<br>2.5 vs 0.5%<br>2.5 vs 0%<br>5.9 vs 2.5%<br>7.4 vs 0%<br>4.9 vs 0%

Table S2. *Cont.*

Yao, 2008 [105]	Pase II study	Everolimus 5 or 10 mg + Octreotide LAR 30 mg	G1/2<br>Metastatic or unresectable carcinoid or pancreatic NET<br>ECOG PS NS	60 pts<br>PanNET = 30	NS	PanNET 50 weeks (12.5 m) (95% CI, 31 to 70 weeks)	Not reached	NA	27%	PanNET PR = 27% (*n* = 8), SD = 60% (*n* = 18)	hyperglycaemia<br>hypertriglycerideemia<br>hypophosphateemia<br>thrombocytopenia<br>leukopenia	9%<br>3%<br>11%<br>3%<br>3%
SUN111 [26,27]	Ph III, Double blind placebo-controlled trial in advanced PanNET	Sunitinib vs Placebo	G1/2<br>Well differentiated PanNETs;<br>No/1 or >/=2 prior Tx lines (121/50 pts);<br>ECOG PS 0–1	171 pts<br><br>Sun vs Pl (86 vs 85 pts)	PFS	Sun vs PL 11.4 m (7.4–19.8) Vs 5.5 m (3.6–7.4 m) #<br>12.6 vs 5.8 m *	Sun vs PL 38.6 m vs 29.1 m (HR 0.73, 95% CI 0.50–1.06, *p* =0.094)^	NA	9%	Sun 72% /*n* = 62/ (CR 2% /*n* = 2/ PR 7% /*n* = 6/ SD 63% /*n* = 54)<br>Pl 60% /*n* = 51/ (CR 0% /*n* = 0/, PR 0% /*n* = 0/, SD 60% /*n* = 51/	Neutropenia<br>Hypertension<br>Abdominal pain<br>Fatigue	<u>Sun vs Pl</u><br>12 vs 0%<br>10 vs 1%<br>5 vs 10 %<br>5 vs 8%
Rinzivillo 2018 [66]	Retrospective	Sunitinib	G1-3 (G3-minority)<br>Progressive panNETs<br>ECOG PS - NS	80 pts	PFS, OS and DCR	10 m	NS	NA	17.5	71.3% CR+PR = 17.5% SD 53.8% (*n* = 43)	ALL	25.4 % - G3<br>1.7 % - G4
Sato, 2018 [67]	Post-marketing study (PMS)	Sunitinib	Well differentiated (90.3%)<br>Other (9.7%)<br>Progressive (unresectable, locally advanced or	61 pts	ORR	NS	NS	165 days	13.7% (95% CI, 5.7–26.3%)	70.6% (95% CI, 56.2–82.5%)	ALL<br>Thrombocytopenia<br>Hypertension<br>Diarrhoea<br>Neutropenia<br>Leukopenia	48.4 % (30 pts)

			metastatic disease)<br>PanNET<br>ECOG PS 0- >/=2									
Raymond, 2018 [68]	Phase IV trial	Sunitinib	well-differentiated<br><br>Progressive, advanced unresectable/metastatic panNETs<br><br>ECOG PS NS	106 pts<br><br>61 – treatment naive<br>45 – later line	PFS	13.2 months (10.9–16.7)<br><br>treatment-naive 13.2 (7.4–16.8)<br><br>previously treated 13.0 (9.2–20.4)	37.8 months (95% CI, 33.0-not estimable)	treatment-naive 19.1 m (10.1 to NA)<br>previously treated 14.7 m (5.5 to 21.9)	24.5% (16.7–33.8)<br><br>treatment-naive 21.3% (11.9–33.7)<br><br>previously treated 28.9% (16.4–44.3)	NS	treatment-naive<br>previously treated	75.4% (46 pts)<br>68.8% (31 pts)

Table S2. *Cont.*

Panzuto, 2014 [71]	Real-world Study	Everolimus	G NS<br>Advanced progressive NETs<br>ECOG PS NS	1069 pts<br>PanNET = 85 pts	Tolerability and efficacy	12 m (All)<br>PanNET similar to Non-PanNET	32 m (All)<br>PanNET similar to Non-PanNET	NS	NS	NS	All 46.1%<br>Pneumonitis 8.3%<br>Thrombocytopenia 7.7%<br>5.3%<br>Anaemia 3.5%<br>Renal failure
The OBLIQUE Study [69]	Phase IV trial	Everolimus	G NS<br>advanced progressive PanNENs<br>ECOG PS 0–2 or not stated	48 pts	Health-related quality of life (HRQoL) from baseline after 6 months treatment	25.1 m<br>(95% CI: 7.8–NR)	NR<br>During the study	NA	NA	55.6%	13 events:<br>• Mucosal inflammation<br>• Diarrhoea<br>• Fatigue<br>• Stomatitis<br>• Arthralgia<br>• Pyrexia<br>• Dry skin
NCT02842749 [70]	Phase IV trial	Everolimus	Well differentiated<br>Adult Patients With Progressive PanNET in China<br>ECOG PS NS	41 pts	Safety and Efficacy	Waiting for results	Waiting for results	Waiting for results	Waiting for results	Waiting for results	Waiting for results

Table S2. *Cont.*

NCT02267967<br>[75,166]	Phase Ib/II, single-arm study	Surufatinib	Grade 1/2<br>Unrespectable/ metastatic NET progressed on or not suitable for standard Tx<br>ECOG PS 0–1	81 pts<br>PanNETvsNonPan-NET<br>42 vs 39 pts **	ORR, safety	PanNET group<br>21.2 m<br>(95% CI 15.9, 24.8)	Not reported	NA	PanNET<br>#19 %<br>*12%	PanNET<br>#91%<br>(PR 19% /*n* = 8/,<br>SD 71% /*n* = 30/,<br>NE~ 7% /*n* = 3/)<br>*93 %<br>(PR 12% /*n* = 5/,<br>SD 81% /*n* = 34/,<br>NE~ 7% /*n* = 3/)	ALL<br><br>Hypertension<br>Proteinuria<br>Hyperuricaemia<br>Diarrhoea	77.8%<br><br>33 %<br>12 %<br>10 %<br>6%
						NonPan-NETgroup<br>13.4 m<br>(95% CI 7.6, 19.3)<br>All NET - 16.9 m			NonPan-NET<br>#15%<br>*10%	NonPan-NET<br>#92%<br>(PR 10% /*n* = 4/,<br>SD 82% /*n* = 32/,<br>NE~ 5% /*n* = 2/)		
NCT01466036<br>[73]	Phase II,<br>Two-cohort study	Cabozantinib	Grade 1/2<br>Progressed carcinoid (CARC) or PanNET<br>ECOG PS 0–1	61 pts<br>PanNETs = 20 pts<br>CARC = 41 pts	ORR	PanNET<br>21.8 m<br>(95% CI, 8.5–32.0 m)<br><br>CARC<br>31.4 m<br>(95%,<br>CI 8.5 m -NR)	NS	NA	PanNET<br>15%,<br>(95% CI 5–36%)<br><br>CARC<br>ORR 15%<br>(95% CI 7–28%)	PanNET<br>PR *n* = 3<br>SD *n* = 15<br><br>CARC<br>PR *n* = 6<br>SD *n* = 26	Hypertension<br>< Phosphate<br>> Lipase or Amylase<br>Diarrhoea<br>Lymphopenia<br>< Thrombocytes<br>Fatigue	13%<br>11%<br>10%<br>8%<br>7%<br>5%<br>5%
TALENT trial<br>[74]	Phase II Prospective multicohort study	Lenvatinib	G1/2<br>Progressed pancreatic or gastrointestinal NET (giNET)<br>ECOG PS NS	111 pts:<br>PanNETs = 55 pts<br>giNETs = 56 pts	ORR by central radiology review	PanNET<br>15.5 m<br>(95%<br>CI 11.3-NR)	PanNET<br>29.2 m<br>(95% CI 23.2-NR)	NA	PanNET<br>42.3%	NS	Hypertension<br>Fatigue<br>Diarrhoea	22%<br>11%<br>11%

Table S2. *Cont.*

SUNEVO<br>(GETNE 1408)<br>[107]	Phase II trial	Evofosfamide + Sunitinib	G1/2<br>Unrespectable or metastatic pancreatic NET naïve for systemic Tx, except SSA<br>ECOG PS 0–1	17 pts	ORR	10.3<br>(2.6–18.0)	NA	18 m<br>(4.2–38.3)	17.6%	CR 5.9% (*n* = 1)<br>PR – confirmed 11.8% (*n* = 2)<br>PR unconfirmed 5.9% (*n* = 1)<br>SD 64.7% (*n* = 11)<br>Not evaluable 5.9% (*n* = 1)	All<br>Neutropenia<br>Hypertension<br>ALT increase<br>Thrombocytopenia<br>Fatigue	52.9%<br>18.8%<br>12.5%<br>12.5%<br>6.3%<br>6.3%
PALBONET<br>[76]	Phase II trial,<br>non-randomised,<br>open-label	Palbociclib	G1/2<br><br>Metastatic PNET progressed on previos Tx	21 pts	ORR	1.9 m<br>(95% CI 0–13)	16.6 m<br>(95% CI 9.3–23.9)	NA	0%	55%<br>CR/PR – 0%<br>SD - 55% (*n* = 11)	Neutropenia<br>Trombocytopenia	5 pts<br>2 pts
Halperin, 2019<br>[77]	Single-arm open-label study	Ziv-Aflibercept	G1-2<br>Metastatic or unable for surgery PanNET,<br>MEN 1 included<br>ECOG PS 0–1	21 pts	ORR	NS	NS	NA	9.5%<br>(2 pts)	NS	Gastrointestinal haemorrhage<br>Proteinuria	1 pts (grade5)<br>5 pts, requiring study discontinuation
Bendell, 2016<br>[108]	Phase II	Bevacizumab +Pertuzumab + Octreotide LAR 30mg	G1-2<br><br>Unresectable or metastatic NET - typical carcinoid or pancreatic islet cell, with documented PD<br>ECOG PS 0–2	43 pts<br><br>PanNET = 11 pts	RR	PanNET<br>5.49<br>(1.1 to 6.5)	PanNET<br>26.4 [2]<br>(3.0 to NA)<br>$	NA	PanNET<br>18%	PanNET<br>91%	ALL<br>SAE:<br>- Nausea<br>- Vomiting<br>-abdominal pain<br>-acute kidney injury<br>- kidney infection<br>LVEF decline<br>Hypertension	13.95%<br><br><br><br><br><br><br>9%<br>26%
Salazar, 2018<br>[64]	Phase II open label, randomised study	BEZ235 or everolimus.	G1-2<br><br>Unresectable or metastatic PanNET, prior systemic Tx </=2<br>ECOG PS 0–2	62 pts<br>BEZ = 31<br>EVE = 31	PFS	BEZ = 8.2m<br>EVE =1 0.8m	6-m OS<br>BEZ = 96,6%<br>EVE = 90,3%	BEZ = 22.9 weeks<br>EVE = 39.4 weeks	Both arms 9.7%	BEZ = 61.3%<br>CR = O<br>PR = 9,7%<br>SD = 51,6%<br>NE = 25,8%<br>EVE =9 0,3%<br>CR = O<br>PR = 9,7%<br>SD = 80,6%<br>NE = 6,5	Diarrhoea<br>Hyperglycaemia<br>Asthenia<br>Stomatitis	BEZ vs EVE<br>83.9 vs 71.0%<br><br>16.1 vs 3.2%<br>16.1 vs 6.5%<br>16.1 vs 3.2%<br>12.9 vs 6.5%

Table S2. *Cont.*

Jin, 2016<br>[78]	Phase II, single arm	Panobinostat	G1-2<br>Metastatic NET<br>ECOG PS 0–2	15 pts<br>PanNET<br>= 5pts	ORR	9.9 m<br><br>(90% CI,<br>4.1–16.9)	47.3 m<br><br>(90% CI, 17.87<br>to not reached)	NA	0%	CR = O<br>PR = 0<br>SD = 47.3%	Fatigue<br>Thrombocytopenia<br>Anorexia<br>Diarrhoea<br>Nausea	G3/4<br>27%<br>20%/7%<br>20%<br>13%<br>13%
COOPERATE-2<br>trial<br>[63]	Phase II Randomised,<br>Open-label, Multicentre<br>Study	Everolimus + Pasireotide<br>LAR<br>Vs Everaolimus Alone	G1-2<br>Advanced progressive PanNET, not<br>requiring somatostatin analog<br>treatment, prior Tx </= 2lines<br><br>ECOG PS	160 pts<br><br>Pas/EVE =<br>79 pts<br>Eve = 81<br>pts	PFS	Pas/EVE<br>16.82 m<br>(12.09 to<br>19.58)<br><br>EVE<br>16.59<br>(11.07 to<br>19.48)<br>HR 0.99<br>(95%CI,<br>0.63-1.64)<br>$p = 0.488$	24 m OS<br>Pas/Eve<br>77.0<br>(65.6 to 85.1)<br><br>EVE<br>71.0<br>(59.3 to 79.9)	NA	PAS/EVE<br>20.3%<br><br>EVE<br>6.2%	PAS/EVE<br>= 77.2%<br>PR = 20.3%<br><br>EVE =<br>82.7%<br><br>PR = 6.2%	SAE all<br><br><br>Hyperglycaemia	Pas/Eve<br>vs<br>EVE<br>41 vs<br>49%<br>37 vs<br>11%
NCT01024387<br>[79]	A phase II multicentre<br>two cohort study	Ganitumab (AMG 479)	G1/2<br><br>Progressed on previous Tx carcinoid<br>or PanNETs<br><br>ECOG PS 0–2	60 pts<br><br>PanNET =<br>30<br>CARC = 30	ORR	6.3 m<br>(95% CI,<br>4.2–12.6)<br><br>PanNET<br>4.2	NR<br>at 12 m<br>66% (95% CI,<br>52–77%)<br>PanNET<br>65% (95% CI,<br>45–80%)	NA	No CR or<br>PR	PanNET<br>SD=31%	ALL pts<br>Hyperglycaemia<br>Neutropenia<br>Thrombocytopenia<br>Infusion reaction	 4%<br>4%<br>4%<br>1%
Phan, 2010<br>[18]	Phase II	Pazopanib in<br>combination with SSAs<br>(octreotide LAR)	G – NS /Low grade/<br>Carcinoid or pancreatic NET<br>ECOG PS - NS	51 pts<br><br>PanNET =<br>29 pts	ResponseToxicity,<br>Survival	PanNET<br>11.7 m	NS	NA	PanNET<br>17%	ALL 72.5%	Anaemia<br>Neutropenia<br>Fatigue<br>Hypertension<br>Diarrhoea	1 pts<br>3 pts<br>3 pts<br>6 pts<br>3 pts

Table S2. *Cont.*

PAZONET [72]	Phase II, Open Label, Uncontrolled Multicentre Trial	Pazopanib	G 1–3<br>Progressive Advanced/Metastatic NET<br><br>ECOG PS 0–1	44 pts<br><br>PanNET = 18 pts	Clinical Benefit Rate at 6m	PanNET 12.8 m	24.1 m<br>For pts previously treated with targeted therapy	ALL 11.3 m (2.0 to 20.6 m)	NS	CR+PR+SD at 6th month<br>73.3% -previously TKIs,<br>66.7% Tx naïve<br>60.0% previously mTOR inhibitors<br>25% - previously TKIs and mTOR	For all pts SAEs<br><br>Hepatotoxicity<br>Asthenia<br>Diarrhoea<br>Hypertension	43.18% (19 pts)<br><br>8%<br>7%<br>4%<br>4%

N—number median PFS—median progression free survival median OS—overall survival DoR—duration of response ORR—objective response rate DCR—disease control rate CR—complete response PR—partial response SD—stable disease SSA—somatostatin analogues pts—patients & central review # Investigator assessment Pl—placebo HR—hazard ratio CI—confidence interval G—grade NS—not stated NA—not applicable Eve—everolimus CHT—chemotherapy PanNET—pancreatic neuroendocrine tumour Sun—sunitinib Tx—therapy CARC—carcinoid NR—not reached PAS—pasireotide SAE—serious adverse events.

Table S3. Summary of studies exploring the role of PRRT.

Study	Design	Treatment	Pts selection (Grade, Prior Therapy, ECOG PS)	N	Primary End Point	median PFS (months)	median OS (months)	DoR (months)	ORR (%)	DCR (CR+PR+SD) (%/n)	G3-4 toxicity	(%)
NETTER 1 [36,81,109]	Phase III open-lable, randomised, international, multicentre trial	177Lu-Dotatate + Octreotide LAR 30 mg vs Octreotide LAR 60 mg	G1/2<br><br>Metastatic midgut NET (excluding PanNETs), progressed on SSA; SSR +<br><br>Karnovski >/=60	229 pts	PFS	Lu vs control<br>Low (LTB) 28.35 vs 11.04 (HR = 0.218, 95% CI 0.120-0.394)<br>Moderate LTB NR vs 8.67 (HR = 0.202, 95% CI 0.077-0.525);<br>High LTB 19.38 vs 5.52 (HR = 0.193, 95% CI 0.079-0.474	NR vs 27.4 m HR 0.398 (95%, 0.207 – 0.766)	NA	Lu 18% (95% CI 10–25%)<br><br>Control 3% (95% CI 0–6%)<br><br><0.001	Lu<br>CR 1% (*n* = 1)<br>PR 17% (*n* = 17)<br>SD NS<br><br>Control<br>CR 0% (*n* = 0)<br>PR 3% (*n* = 3)<br>SD NS	Neutropenia<br><br>Thrombocytopenia<br><br>Lymphopenia<br><br>Nausea<br><br>Abdominal pain	Lu vs control<br><br>1 vs 0%<br><br>2 vs 0%<br><br>9 vs 0%<br><br>7 vs 1%<br><br>3 vs 5%
T.Brabander, 2017 [37]	Retrospective study	177Lu-DOTATATE	Mainly G1–2<br><br>Pts with bronchial or GEP NET, SSR+ treated with >/=600miCi Before 2013<br><br>Karnovski >/=50	443 pts<br><br>PanNET = 133 pts	Safety, Efficacy, Survival	29 m (95% CI, 26–33 m)<br><br>PanNET 30 m	63 m (95% CI, 55–72 m)<br><br>PanNET 71 m	NA	ALL 39%<br><br>PanNET 54% (*n* = 72)	ALL<br>CR 2% (*n* = 9)<br>PR 37% (*n* = 165)<br>SD 43% (*n* = 192)<br>NE 5% (*n* = 24)<br>PanNET<br>CR 5% (*n* = 6)<br>PR 50% (*n* = 60)<br>SD 30% (*n* = 40)<br>NE 3% (*n* = 4)	Leukaemia<br><br>Myelodysplastic syndrome	0.7%<br><br>1.5%

Table S3. *Cont.*

Garske-Román,U, 2018 [38,43]	Prospective observational study	177Lu-DOTATATE applying systematic, individualised dosimetry of the kidney and bone marrow	G1–3 / unknown<br><br>Metastatic NET, progressing on or not suitable for standard Tx, SSTR+<br><br>ECOG PS NS	200 pts<br><br>Duodenal or PanNET = 49 pts	NS	PanNETs<br>27 m (95% CI 17–33 m)<br>ALL<br>Pts with CR/PR 31 m, (95% CI 23–35 m),<br>In pts with SD 28 m, (95% CI 21–31 m),<br>Not significant	PanNETs<br>42 (31–NR)<br>Pts with CR/PR 60 m, (95% CI 43 m- NR)<br>In pts with SD<br>42 m,<br>(95% CI 34–52 m),<br><br>p = 0.004	NA	PanNETs 45% (n = 22)	PanNET =94% (n = 46)<br>CR 2% (n = 1)<br>PR 43% (n = 21)<br>SD 49% (n = 24)	acute leukaemia<br><br>chronic leukaemia<br><br>marrow toxicity<br><br>kidney toxicity-	1.5%<br><br>0.5%<br><br>15%<br><br>0.5
Sansovini, 2017 [39,43]	Phase II study	177Lu-DOTATE<br>Full Activity (FA) vs Reduced Activity (RA)	G1/2<br><br>Unresectable/ metastatic PanNET, progressed on prior Tx, SSR+<br>ECOG PS NS	60 pts<br><br>FA = 28 pts<br>RA = 32 pts	NS	All = 29 m (20-54 m)<br><br>FA= 53.4 m<br>RA =21.7 m<br><br>*p* =0.353	All=Not reached<br><br>FA = Not Reached<br>RA = 63.8 m<br><br>*p* = 0.007	NA	30% (*n* = 30	All = 82% (*n* = 49)<br>CR 7% (*n* = 4)<br>PR 23% (*n* = 14)<br>SD 52% (*n* = 31)<br>FA = 85.7 %<br>RA = 78,1%	FA<br>Anemia<br>Leukopenia<br>Thrombocytopenia<br>RA<br>Anemia<br>Leukopenia<br>Thrombocytopenia<br>*from Ramage	0<br>0<br>0<br><br>0<br>0<br>0
Dumont, 2015 [40,43]	Phase II study	90Y-DOTATOC vs. 90Y-DOTATOC plus 177Lu-DOTATOC	G NS<br>Metastatic gastrinomas with baseline PD<br>ECOG PS NS	36 pts<br><br>Y = 30 pts<br>Y= Lu = 6 pts	Survival	NS	40.1 m	NA	33,3% (*n* = 12)	NS	Heamatological Toxicity<br>Renal toxicity	2.8%<br><br>5,6%
Bertani, 2016 [41,43]	Prospective trial	90Y-DOTATOC or 177Lu-DOTATOC or both +/- initial primary tumour resection (PTR)	G1/2<br><br>PanNET with unresectable liver mets, not suitable for radical surgery, without previous Tx<br>ECOG PS NS	Total = 94 pts<br>Evaluable = 90 pts	RR,<br>OS,<br>PFS	PAnNET 36 (24-44)<br>With vs without PTR 70 vs. 30 m<br>*p* = .002	PanNET 76 (64-104 m)<br><br>With vs Without PTR<br>112 vs. 65m<br>*p* = .011	NA	26% (*n* = 23)	All = 68% (*n* = 61)<br><br>CR 0%<br>PR 26% (*n* = 23)<br>SD 42% (*n* = 38)	Not sated	

Table S3. *Cont.*

Imhof, 2011<br>[43,167]	Phase II single-center open-label trial	90Y-DOTATOC	G-NS<br>Metastatc NEN, with PD on baseline CT<br><br>ECOGPS NS	1109 pts<br>PanNET = 342 pts<br>NF PanNET = 295<br>Gastrinoma = 25<br>Insulinoma = 8<br>Glucagonoma = 8<br>VIPoma = 4<br>ACTHoma = 2	OS,<br>renal toxicity grade 4–5 Tx response.	NS	NF PanNET = 60 m<br>GAstrinoma = 32<br>Insulinoma = 17<br>Glucagonoma = 39<br>VIPoma = 40<br>ACTHoma = 5	NA	PanNET 47% (*n* = 161)<br>NF PanNET 49% (*n* = 145)<br>Gastrinoma 20% (*n* = 5)<br>Insulinoma 38% (*n* = 3)<br>Glucagonoma 50% (*n* = 4)<br>VIPoma 75% (*n* = 3)<br>ACTHoma 50% (*n* = 1)	**CR -**<br>NF PanNET 0.7% (*n* = 2)<br><br>Gastrinoomas 12% (*n* = 3)<br><br>Others - Not spesified	All pts<br><br>Haematological toxicity<br><br>Renal impairment	12.8%<br><br>9.2%
Rogowski, 2016<br>[43,44]	Phase II study	90Y-DOTATATE	G1/2<br>Pancreatic and small bowell tumours, SSRT+ , baseline PD or biochemical/clinical symptoms<br>ECOG PS NS	67 pts<br><br>PanNET = 30 pts	OS	PanNET<br>25 m<br>(21–33 m)	PanNET<br>42 m<br>(34–48 m)	NA	PanNET<br>39% (*n* = 12)	PanNET - NS<br>CR 0%<br>PR 39% (*n* = 12)<br>SD NS	NS	
Hamiditabar, 2017<br>[43,45]	Expanded access trial	177Lu-DOTATOC	G NS<br>NET with baseline PD, SSTR+<br>ECOG PS NS	132 pts<br><br>PanNET = 48 pts	Response – PFS, OS, radiologic, biochemical, and clinical response	NS	NS	NA	PanNET = 13% (*n* = 6)	PanNET = 50% (*n* = 24)<br>CR 0%<br>PR 13% (*n* = 6)<br>SD 38% (*n* =18)	ALL<br>Haematological toxicity<br>Hepatotoxicity	N = 16<br><br>N =3
Horsch, 2016<br>[43,45]	Retrospective	90Y-DOTATOC or 177Lu-DOTATOC or both	Most of pts G1/G2<br>Advanced or metastatic NEN with baseline PD, SSTR+<br>ECOG PS OA	445 pts<br><br>PanNET = 172 pts	PFS,<br>OS,<br>Side effects	PanNET<br><br>39 m<br>(29–49 m)	PanNET<br><br>53 m<br>(37–69 m)	NA	NS	ALL<br><br>CR 5.6%<br>PR 22.4%<br>SD 47.3%	ALL<br><br>bone marrow and kidney toxicity	0.2–1.5%
Baum, 2018<br>[43,46]	Retrospective	90Y-DOTATOC or 177Lu-DOTATOC or both	G1–3<br>NEN with baseline PD, SSR+<br>ECOG PS NS	1,048 pts<br><br>PanNET = 384 pts	PFS,<br>OS	PanNET<br>20 m<br>(17–23 m)	PanNET<br>44 m<br>(38–50 m)	NA	NS	**NS**	ALL<br>Leucopenia<br>Thrombocytopenia<br>Anaemia<br>Chronic kidney disease	N = 8<br>N = 9<br>N = 17<br>N = 14

Table S3. *Cont.*

Erasmus MC Clinical Study [82]	Phase I/II single arm	177Lu-Oxodotreotide	G – NS GEP-NET or bronchial carcinoid, SSTR + Karnofsky PS ≥50	1214 pts Dutch=811 Non-Dutch=403 *The efficacy analysis was done only on Dutch GEP-NET=558 pts Dutch PanNETs 197 pts (35.3%)	RR	Dutch PanNET= 30.5 m<br><br>Dutch PanNET pts progressive at baseline= 35.6 m	Dutch PanNET= 70.8 m<br><br>Dutch PanNETpts progressive at baseline = 80.7 m	Dutch PanNET= 16.3 m (95%CI 12.1–21.8)	Dutch PanNET = 60.9% (*n* = 81) (95% CI 52.1–69.2%)	Dutch PanNET = 96.2%<br><br>CR 5.3% (*n* =7)<br>PR 55.6% (*n* =74)<br>SD 35.3% (*n* =47)	Dutch GEP-NET<br>Leucopenia<br>Neutropenia<br>Lymphopenia<br>Thrombocytopenia<br>Anaemia<br>> GGTP<br>>ALAT	2.4%<br>1.1%<br>29.6%<br>1.7%<br>1.1%<br>18.9%<br>2.8%

N—number median PFS—median progression free survival median OS—overall survival DoR—duration of response ORR —objective response rate DCR—disease control rate CR—complete response PR—partial response SD—stable disease G—grade SSA—somatostatin analogues pts—patients LTB—liver tumour burden NR—not reached NA—not applicable HR—hazard ratio CI—confidential interval Lu—Lutetium NS—not stated SSTR—somatostatin receptor FA—full activity RA—reduced activity Y—Yttrium PTR—primary tumour resection FA—functioning NEN—neuroendocrine neoplasms.

Table S4. Summary of studies exploring the role of chemotherapy.

Study	Design	Treatment	Pts selection (Grade, Prior Therapy, ECOG PS)	N	Primary End Point	median PFS (months)	median OS (months)	DoR (months)	ORR (%)	DCR (CR+PR+SD) (%/n)	G3-4 toxicity	(%)
E2211 trial [31]	phase II two-arm, randomised trial	Temozolomide(t) vs Temozolomide and capecitabine (TemCap)	G1/2<br><br>Metastatic or unresectable PanNETs, progressed within preceding 12 months, and no prior T, C, DTIC, or 5-FU<br><br>ECOG PS - NS	144 pts<br><br>T = 72 pts<br>TemCap = 72 pts	PFS	T = 14.4 m<br>TemCap = 22.7 m<br>(HR = 0.58, *p* = 0.023)	T = 38 m<br>TemCap = Not reached<br>(HR = 0.41, *p* = 0.012)	T = 9.7 m<br>TC = 12.1 m	NA	T:<br>CR 2.8%<br>PR 25%<br>SD<br><br>TemCap:<br>CR 0%<br>PR 33.3%<br>SD	All<br>Neutropenia<br>Lymphopenia<br>Thrombocytopenia<br>Nausea<br>Vomiting<br>Diarrhoea<br>Fatigue	T vs TemCap<br>22 vs 44%<br>4 vs 13%<br>4 vs 5%<br>13 vs 8%<br>0 vs 8%<br>0 vs 8%<br>0 vs 8%<br>1 vs 8%
De Mestier, 2019 [32]	Large multicenter series, Retrospective	Temozolomide(T) vs Temozolomide and capecitabine (TemCap)	G1-3<br>Locally advanced or metastatic PanNET,<br><br>ECOG PS 0–2	138 pts	PFS	T = 21.4 m<br>TemCap = 19.8 m<br><br>HR 0.96, 95% CI 0.63–1.47, *p* = 0.84)	T = 47.6 m<br>TemCap = 75.2 m<br><br>(HR 0.66, 95% CI 0.37–1.19, *p* = 0.16)	NA	T vs TemCap<br>34.2% vs 51%	T 73.7 :<br>CR 5.3%<br>PR 28.9%<br>SD 39.5%<br>Vs<br>TemCap 87%:<br>CR 2%<br>PR 49%<br>SD 36%	Nausea/Vomiting<br>Diarrhoea<br>Neutropenia<br>Thrombocytopenia<br>Asthenia<br>HFS	T vs TemCap<br>2.6 vs 2.1%<br>0 vs 2.1%<br>5.3 vs 6.4%<br>13.2vs12.8%<br>7.9 vs 0%<br>0 vs 2.3%
Campana, 2018 [33]	A multicenter, international retrospective analysis	Temozolomide(T) or Temozolomide and capecitabine (TemCap) in MGMT (+) vs MGMT (−)	G1-2 or NEC<br>Metastatic NEN (locally advanced 1 pts), with PD before treatment mainly (93 pts), who had or no previous Tx (75 vs 20%)<br>ECOG PS - NS	95 pts<br>PanNET = 43 pts<br>T = 31.9%<br>TemCap = 68.1%	Correlation between OR and MGMT promoter status	ALL – 10 m<br><br>PanNET= 13 m	ALL = 33 m<br><br>PanNET = 35 m	NA	MGMT + 51.8 %<br><br>MGMT – 17.7%	ALL<br>PR = 27.4%<br>SD = 44.1%	NS	

Table S4. *Cont.*

Kunz, 2016 [91]	2separate prospective phase II trials	FOLFOX + Bevacizumab (B) study and CAPOX/Bevacizumab (B) study	G NS Advanced NET ECOG PS NS	76 pts FOLOFOX/B = 36 pts PanNET = 12 CAPOX/B = 40 pts PanNET = NS	RR at 12 cycle (FOLFO X/B) PFS (CAPO X/B) and toxicity - both	FOLFOX/B PanNET = 21m CAPOX/B PanNET = NS ALL = 16.7 m	NS	NA	FOLFOX/B PanNET = 41% CAPOX/B PanNET = NS All 18%	**NS**	**NS**	
BETTER trial [28]	Phase II trial open-label, non-randomised, two-group study	Bevacizumab + 5-FU and Streptozocin Minimum 6 cycles	G1–2 Progressive metastatic PanNET ECOG PS 0–1 (97%)	34 pts	PFS	At 24 m: 23.7 m (95% CI: 13.1-NR)	At 24 m: 88%	NA	56%	CR = 0 PR = 56% (n = 19) SD = 44% (n = 15)	Hypertensio Abdominal pain Thromboembolic events	21% 12% 9%
Chan, 2013 [94]	Phase 1/2 prospective study	Temozolomide + Everolimus 5mg (cohort1) or 10mg (cohort2)	G1–2 Metastatic or locally unresectable pancreatic NET ECOG PS 0–2	43 pts Cohort1 = 7 pts Cohort2 = 36 pts	RR	15.4 m (95% CI, 9.4–20.4 m)	Not reached	NA	40%	CR=0 PR = 40% (n =16) SR = 53% (n = 21)	Lymphopenia Thrombocytopenia Pneumonitis	44% 16% 0%
Dilz, 2015 [29]	Retrospective	STZ/5-FU	G1–3 Advanced PanNET, Baseline progression was evident in 74% ECOG PS NS	96 pts	ORR, TTP, OS	NS	54.8 m	NA	42.7%	CR = NS PR = NS SD = 40.6%	NS	
Moertel, 1992 [30]	Phase III	Chlorozotocin vs. Streptozotocin + 5-FU vs. Doxorubicin +Streptozotocin	G – NS Unresectable or metastatic islet-cell cancer ECOG PS 0–3	102 Chlorozotocin = 33 pts STZ/5-FU = 33 pts STZ/Doxorubicin = 36 pts	NS	CHL 17 m STZ/5-FU 14 m STZ/Doxo 18 m	Chl 18 m STZ/5-FU 16.8 m STZ/Doxo 26.4 m	NA	Chl 30% STZ/5-FU 45% STZ/Doxo 69%	NS	Chl vs STZ/5-FU vs STZ/Doxo Vomiting: 2 vs 41 vs 20 pts Leucopenia: 14 vs 25 vs 5 pts Thrombocytopenia: 6 vs 6 vs 0 pts Chronic renal insufficiency: 7 vs 7 vs 4 pts	
Delaunoit, 2004 [85]	Retrospective	Doxorubicin and Streptozotocin	Well-differentiated pancreatic endocrine carcinomas ECOG PS NS	45 pts	NS	16 m	2-year survival rate 50.2% 3y survival rate 24.4%	NA	36%	60%	Neutropenia Vomiting	24% 13%

Table S4. *Cont.*

Kouvaraki , 2014 [86]	Retrospective	Streptozotocin, Doxorubicin and 5-FU	G - NS<br>Locally advanced or metastatic NEC<br>ECOG PS - NS	84 pts	ORR, PFS, OS	18 m	37 m	Na	39%	89% (PR = 39% SD = 50%	All<br>Leucopenia<br>Thrombocytopenia<br>Fatigue<br>Mucositis	23%<br>10.7%<br>1.1%<br>4.7%<br>4.7%
Turner, 2010 [87]	Retrospective	5-FU, Cisplatin and Streptozotocin	G 1–3 (PanNETs G1/2 36, G3 9 pts)<br>Metastatic or locally advanced neuroendocrine tumours, chemo naive<br>ECOG PS 0- >/=2	82 pts<br><br>PanNET = 49 pts	NS	ALL 9.1 m	All 31.4 m	NA	PanNET = 38%	PanNET = 86%	For All pts<br>Nausea<br>Vomiting<br>Neutropenia	 12 pts<br>14 pts<br>23 pts
Ramanathan, 2001 [88]	Phase II	Dacarbazine	G NS<br>Advanced pancreatic islet cell tumours, with progressive symptoms or evidence of rapidly advancing disease<br>ECOG PS 0–3	50 pts	RR, Safety, Surviva, DoR	NS	19.3 m	10 m (4-28 m)	34%	CR = 8%<br>PR = 26%<br>SD = NS<br>NE = 3.8%	All<br>Haematological<br>Vomiting	30%
Kulke, 2006 [92]	Phase II	Temozolomide and Thalidomide	G1-2 (1 pts with poorly diff. tumours)<br>Metastatic carcinoid, phaeochromocytoma, pancreatic NET<br>ECOG PS 0–1	29 pts<br>PanNET = 11 pts (38%)	RR	NS	All<br>2y survival 61%	ALL = 13.5 m	PanNET 45%	PanNET 93%	In all pts<br>Lymphopenia<br>Neutropenia<br>Diarrhoea<br>Infections	 20 pts/69%<br>2 pts/6%<br>4 pts/14%<br>5 pts/17%

Table S4. *Cont.*

Strosberg, 2011 [34]	Retrospective	Temozolomide and Capecitabine	G1–2<br>Pts with metastatic PanNETs who have not received systemic therapy<br>ECOG PS	30 pts	RR, Surviva, Safety	18 m	2y-survival 92%	NA	70%	97%<br>CR = 0<br>PR = 70%<br>SD = 27%	All	14%
Chan, 2012 [93]	Phase II	Temozolomide and Bevacizumab	G1–3<br>Metastatic or locally unresectable NETs<br>ECOG PS </=2	35 pts<br><br>PanNET = 15 pts (44%)	RR	PanNET<br>14.3 m	PanNET<br>41.7m	NA	PanNET<br>33.3%	PanNET<br>87%<br>CR = 0<br>PR = 15%<br>SD = 65%	For all pts<br>Lymphopenia<br>thrombocytopenia	 53%<br>18%
Venook, 2008 [89]	Phase II	5-Fu, Oxaliplatin and Bevacizumab	G1–2<br>Carcinoid tumours (CARC), pancreatic NET (PanNET), or platinum-refractory poorly differentiated NET with clinical or radiologic progression<br>ECOG PS 0–1	13 pts<br><br>PanNET = 6 pts	Efficacy and safety	NS	NS	NA	20%	PanNET 100%<br>CR = 0<br>PR = 33%<br>SD = 67%	abdominal pain<br>anaemia neutropenia<br>FN<br>fatigue<br>ascites<br>gastrointestinal<br>haemorrhage<br>hypertension<br>thrombocytopenia<br>diarrhoea<br>neuropathy	15.4%<br>15.3%<br>30.8%<br>7.6%<br>38.4%<br>7.6%<br>7.6%<br>23%<br>7.6%<br>7.6%<br>15.3%
Kunz, 2010 [90]	Phase II	Capecitabine, Oxaliplatin and Bevacizumab	G1–3<br>Metastatic or unrespectable NET<br>ECOG PS - NS	40 pts<br>PanNET = 20 pts	PFS, toxicity	ALL 13,7m	NS	NA	PanNET 30%	ALL 94%	NS	
De Mestier, 2019 [35]	Retrospective	TemCap vs 5-FU/DTIC (dacarbzaine)	G1–3 (majority G1–2 pancreatic or small-intestine advanced NET	247pts<br>94 - 5FU/DTIC 153 – TemCap<br>PanNET = 82.3%	Toleran ce<br>RR<br>PFS	5FU/DTIC 13.9 m<br>TemCap<br>18.3 m<br>*p* = 0.86	NS	NA	TemCap<br>38.3 m<br>5FU/DTIC<br>39.2%<br>*p* = 0.596	NS	TemCap = 24.7%<br>5FU-/DTIC = 8.5%	

N—number median PFS—median progression free survival median OS—overall survival DoR— duration of response ORR—objective response rate DCR—disease control rate CR—complete response PR—partial response SD—stable disease G—grade T—temozolomide TemCap—tomozolomide and capecitabine pts—patients NA—not applicable HR—hazard ratio CI—confidence interval MGMT—O6-methylguanine DNA methyltransferase NS—not stated FOLFOX—5-Fluoruracil + oxaliplatin CAPOX—capecitabine + oxapliplatn STZ—streptozotocin 5-

FU—5- fluorouracil TemCap—tomozolomide and capecitabine 5-FU/DTIC—5- fluorouacil and dacarbazine CARC—carcinoid Tx—therapy NET—neuroendocrine tumour NEC—neuroendocrine carcinoma.

Table S5. Summary of studies exploring the role of immunotherapy.

Study	Design	Treatment	Pts selection (Grade, Prior Therapy, ECOG PS)	N	Primary End Point	median PFS (months)	median OS (months)	DoR (months)	ORR (%)	DCR (CR+PR+SD) (%/n)	G3–4 toxicity (%)
KEYNOTE -028 [49]	Phase Ib multicohort study	Pembrolizumab	G1/2<br><br>Advanced carcinoids or pancreatic NET, progressive on standard Tx, PD-L1–positive (>1%)<br>ECOG PS 0–1	41 pts<br>PanNET = 16 pts	ORR per RECIST v1.1 by investigato r review	PanNET = 4.5 m	PanNET = 21 m	PanNET 9.2 m (for responder is ongoing response of 17.6 m)	PanNET 6%	PanNET 94%<br>CR = 0<br>PR = 6% (n = 1)<br>SD = 88% (n = 14)	NS
KEYNOTE-158 study [133]	A phase II basket study in selected group of pts with advanced solid tumours	Pembrolizumab	Well/moderately-differentiated NET of the lung, appendix, small intestine, colon, rectum, or pancreas Progression or intolerance to ≥ 1 line of standard therapy<br>ECOG PS 0–1	1032 pts<br><br>NET = 107 pts<br>PanNET- number not mentioned	ORR by central review	ALL 4.1 m (95% CI 3.5–5.4)<br><br>ALL 6 m PFS rate -38.2%.	Not reached (95% CI 18.8-not reached)<br><br>6 m OS rate - 84.6%.	Not Reached	ALL 3.7%	CR = 0<br><br>PR = 4 (3 PanNET, 1 giNET)<br><br>SD = 61	20.6%

Table S5. *Cont.*

NCT02955069 [50]	A phase 2, multi-centre study	Spartalizumab	well- & poorly-differentiated Non-functional NEN, progressed on prior Tx, regardless of PD-L1 ECOG PS - NS	116 pts<br><br>PanNET = 33 pts<br>GI NET = 32 pts<br>T NET = 30 pts<br>GEP NEC = 21 pts	ORR by central review	NR	NA	NA	3% in PanNET<br><br>7.4% in well-diff NET<br><br>4.8% in poorly-diff GEP NEC	T NET 73.3% (PR = 20.0% + SD = 53.3% + Unknown = 10.0%)<br>PanNET 57.6% (PR = 3.0% + SD = 54.5% + Unknown = 3.0%)<br>GI NET 59.4% (PR = 0% + SD = 59.4% + Unknown = 6.3%)<br>ALL NET 63.2% (PR = 7.4% + SD = 55.8% Unknown = 6.3%)<br>NEC 19.0% (PR = 4.8% + SD = 14.3% + Unknown = 14.3%)	>/= 2.5%<br>Abdominal pain;<br>Back pain;<br>Anaemia;<br>Dyspnoea;<br>Hypertension.

N—number median PFS—median progression free survival median OS—overall survival DoR—duration of response ORR—objective response rate DCR—disease control rate CR—complete response PR—partial response SD—stable disease G—grade Tx—therapy pts—patients NS—not stated NA—not applicable NR—not reached PD L1—programme death ligand 1 T NET—thoracic neuroendocrine tumours GI NET—gastrointestinal neuroendocrine tumours NET—neuroendocrine tumour NEC—neuroendocrine carcinoma GEP NEC—gastro-entero-pancreatic neuroendocrine carcinoma.

© 2020 by the authors. Licensee MDPI, Basel, Switzerland. This article is an open access article distributed under the terms and conditions of the Creative Commons Attribution (CC BY) license (http://creativecommons.org/licenses/by/4.0/).
